# Formal uniforms as an environmental constraint on NEAT and adolescent obesity: a cross-sectional study in boarding schools

**DOI:** 10.3389/fpubh.2026.1817702

**Published:** 2026-06-12

**Authors:** Yuan Wang, Rubing Lin, Wei Zhang, Yantong Liu

**Affiliations:** 1 School Uniform Management Professional Committee, China Association for Campus Management, Beijing, China; 2 Department of Orthopedics, Shenzhen Children's Hospital, Shenzhen, China; 3 Department of Clinical Laboratory, Peking University Shenzhen Hospital, Shenzhen, China; 4 Department of Computer and Information Engineering, Kunsan National University, Gunsan, Republic of Korea

**Keywords:** adolescent obesity, environmental constraint, gender disparity, non-exercise activity thermogenesis (NEAT), school uniforms, sedentary behavior

## Abstract

**Background:**

The rising prevalence of obesity in adolescents is commonly attributed to a decrease in moderate to vigorous physical activity (MVPA), but the role of environmental constraints on thermogenesis from non-exercise activities (NEAT) remains under-explored. School uniforms are a daily necessity and may be associated with physical or perceived barriers to spontaneous low-intensity movement. This study investigated the relationship between type of school uniform (formal versus athletic) and body mass index (BMI) and examined whether NEAT-related behaviors showed a statistical mediation pattern in a gender-specific manner.

**Method:**

A cross-sectional study was conducted in four 100 percent boarding secondary schools in China. A total of 1,068 adolescents were stratified by mandatory uniform type. NEAT-related behaviors were assessed using an adapted self-reported questionnaire and compared with accelerometry-derived metrics in a sedentary validation sub-sample (*n* = 108). Mediation analyses and moderated mediation analyses were conducted to examine the uniform type, NEAT-related behaviors, and BMI associations.

**Results:**

Formal uniforms were significantly associated with higher BMI in women (*Δ* = 0.7 kg/m^2^, *p* < 0.01) but not in men (*p* > 0.05). Mediation analyses suggested that this association was statistically mediated by lower NEAT-related behaviors [β_indirect = 0.123, 95% CI (0.054, 0.208)], accounting for 60.3% of the total effect. Exploratory subgroup analyses showed a larger uniform-BMI difference among students in the lowest NEAT-related activity decile: in the lowest decile of sedentary behavior, the difference in body mass index between homogeneous groups widened from a negligible amount in highly active girls to a larger observed difference of 1.3 kg/m^2^ (*p* < 0.01).

**Conclusion:**

Formal school uniforms were associated with lower NEAT-related movement and higher body mass index in adolescent females in this study population. This relationship was more pronounced among students with lower baseline activity levels, suggesting that restrictive clothing may act as an environmental constraint on spontaneous low-intensity movement. These findings suggest that movement-friendly school uniform designs may warrant further investigation in residential school settings.

## 1 Introduction

The escalating prevalence of adolescent obesity remains a formidable global public health challenge, with far-reaching implications for long-term metabolic health and psychological well-being (1, 2). While traditional interventions have predominantly emphasized the enhancement of Moderate-to-Vigorous Physical Activity (MVPA), contemporary epidemiological evidence suggests that Non-Exercise Activity Thermogenesis (NEAT)—the energy expenditure associated with daily spontaneous movements such as standing, walking, and fidgeting—plays an important role in maintaining cumulative energy balance (3–5). NEAT represents a variable and context-dependent component of daily energy expenditure, and its contribution may differ substantially according to lifestyle, school routines, and environmental constraints (6). However, within the structured micro-environment of boarding schools, the structural factors associated with lower NEAT-related behaviors remain under-researched. Among these, the school uniform, a mandatory physical artifact, represents a potentially significant yet overlooked constraint on adolescent daily activity patterns (7, 8).

School uniforms are historically designed to foster social cohesion, minimize socioeconomic disparities, and maintain institutional discipline (9). Nevertheless, from a physiological perspective, the design and material properties of different uniform types may exert different behavioral implications through physical restriction and thermal discomfort. Traditional formal uniforms—typically characterized by non-elastic fabrics, shirts, blazers, and restrictive skirts or trousers—may subtly inhibit the range of motion and discourage spontaneous physical play (10, 11). Drawing on the environmental press and person-environment fit perspective, which proposes that behavioral adaptation emerges from the interaction between individual capacity and environmental demands (12), school uniforms may be conceptualized as a daily environmental demand within the school micro-environment. In this context, clothing-related physical resistance or perceived postural discomfort may be associated with more sedentary behavioral adaptation during routine school activities (13, 14). This chronic reduction in daily low-intensity movement can lead to a positive energy balance and subsequent increases in Body Mass Index (BMI), particularly in high-exposure environments such as 24-h boarding schools where dietary intake is homogenized (15).

Crucially, the impact of school uniforms on physical activity appears to exhibit a profound gender-based asymmetry. Existing literature suggests that female adolescents face greater clothing-related physical or perceived barriers when wearing traditional skirts or tailored formal uniforms compared to their male counterparts (16, 17). This disparity is not merely mechanical; it is deeply rooted in socio-cultural norms surrounding “modesty” and “feminine decorum.” Female students often report that restrictive clothing, particularly skirts, limits their ability to run, climb, or even sit comfortably during breaks, leading to a state of more sedentary behavioral tendencies (18, 19). Previous studies have emphasized that clothing functionality is a critical prerequisite for physical activity participation among girls (20). Consequently, understanding how uniforms influence BMI through the mediation of NEAT is vital for addressing gender-stratified obesity risks.

Students with lower baseline NEAT-related activity may be more sensitive to additional environmental constraints. Therefore, we treated the lowest activity decile analysis as an exploratory subgroup analysis rather than as a confirmatory test of a separate theoretical framework. For “sedentary-prone” students who inherently lack exercise habits, NEAT constitutes the primary avenue for energy outflow (21). In this vulnerable sub-population, even minor physical constraints imposed by clothing may serve as the may be more sensitive to additional environmental constraints (22). Despite the theoretical plausibility of this link, most cross-sectional studies rely on self-reported questionnaires, which are prone to recall bias and social desirability effects, thus complicating the precise quantification of NEAT (23). To address these methodological limitations, the present study incorporates wearable accelerometry (smartwatches) to validate subjective reports against an objective accelerometry-based reference in a sub-sample, providing supplementary objective activity information (24).

Despite the growing body of research on adolescent health, few studies have explored the specific pathways connecting uniform type, NEAT, and BMI within the strictly controlled environment of a 100% boarding school. This study aims to examine the association between school uniform types and BMI among boarding adolescents, with a specific focus on the mediating role of NEAT.

**Figure 1:**
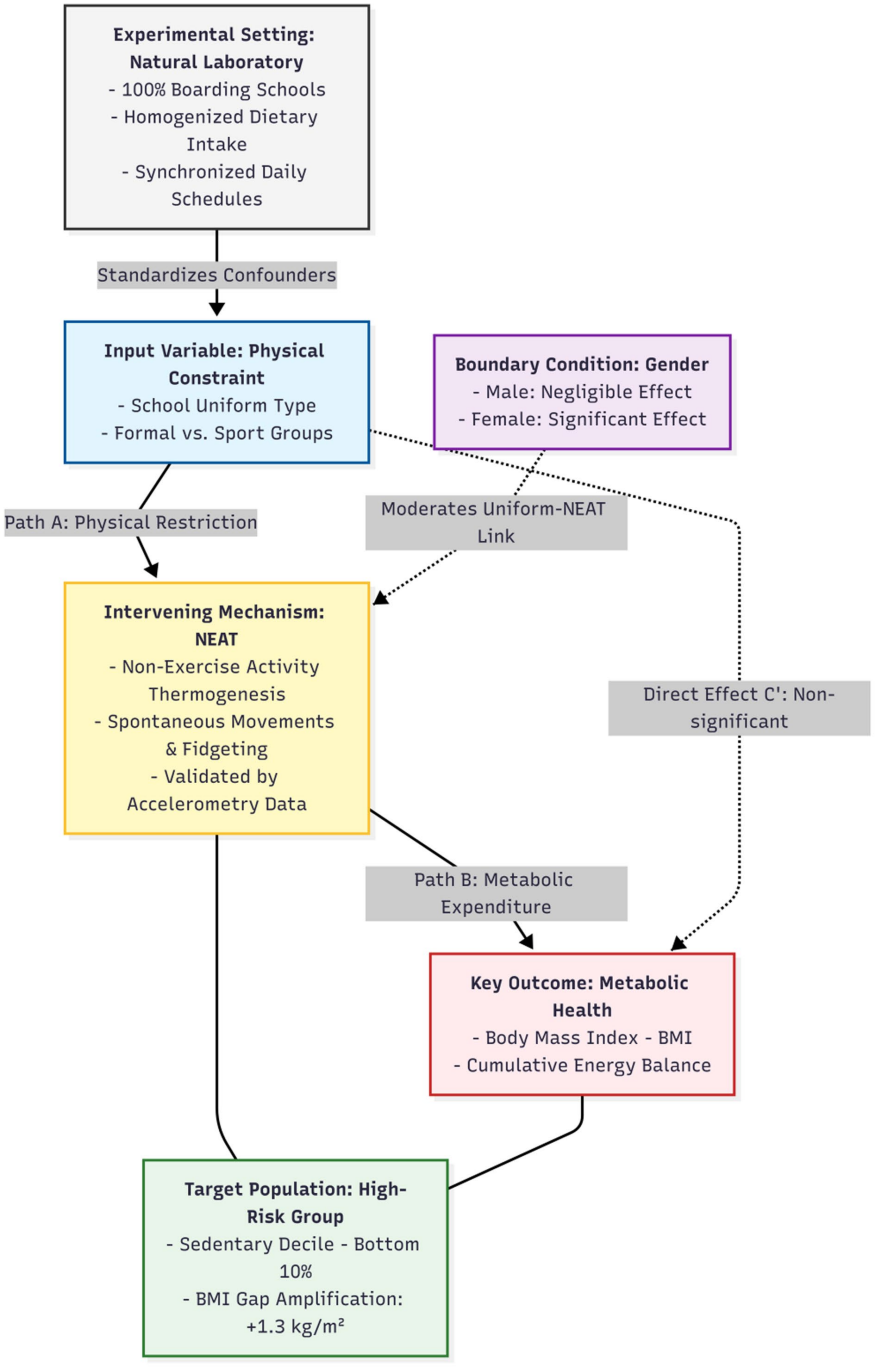
Conceptual framework of the cross-sectional statistical mediation model.

We hypothesized that formal uniforms would be associated with higher BMI, particularly among female students, and that NEAT-related behaviors would show a statistical mediation pattern in the association between uniform type and BMI. In addition, we conducted an exploratory subgroup analysis to examine whether the uniform-BMI association was larger among students in the lowest NEAT-related activity decile (see Figure 1).

## 2 Methods

### 2.1 Study design and setting

This study employed a cross-sectional, multi-center design to investigate the relationship between school uniform types, non-exercise activity thermogenesis (NEAT), and Body Mass Index (BMI). To maximize internal validity and control for confounding lifestyle factors, the study was conducted exclusively in four large-scale, 100% boarding middle schools in China. This setting helped reduce variability in diet, commuting, and daily schedules, although residual confounding could not be eliminated.

### 2.2 Participants and recruitment

A total of 1,068 adolescents (*N* = 1,068) were recruited for this cross-sectional study using a stratified cluster sampling method from four residential middle schools in China. To minimize confounding environmental noise, the sampling was restricted to schools with a 100% boarding rate, where students reside on campus from Monday morning to Friday afternoon. The initial recruitment targeted 1,200 students; however, after excluding individuals with chronic metabolic disorders, physical disabilities that impede movement, or incomplete questionnaire responses, the final effective sample consisted of 1,068 participants (Sport Uniform group, *n* = 542; Formal Uniform group, *n* = 526).

Consistent with the anthropometric profiles detailed in Table 1, the cohort showed broadly comparable demographic characteristics. The mean age of the participants was 16.4 ± 0.8 years, with no statistically significant age difference between the Sport group (16.3 ± 0.9) and the Formal group (16.5 ± 0.8; *t* = 1.24, *p* = 0.215). Gender distribution was balanced, with 556 males (52.1%) and 512 females (47.9%), and no significant variation in gender proportions across uniform types was observed (*χ*^2^ = 0.08, *p* = 0.775). Anthropometric baselines further confirmed that height was comparable (169.5 ± 8.1 cm vs. 168.9 ± 8.6 cm; *p* = 0.241), although weight and BMI showed small but statistically significant differences (*p* = 0.048 and *p* = 0.042, respectively). These baseline differences were considered when interpreting subsequent association and mediation analyses.

**Table 1:** Demographic and anthropometric characteristics of participants by uniform type.

Variables	Total (*N* = 1,068)	Sport uniform (*n* = 542)	Formal uniform (*n* = 526)	*t*/*χ*^2^	*p*-value
Age (years)	16.4 ± 0.8	16.3 ± 0.9	16.5 ± 0.8	1.24	0.215
Gender (*n*, %)				0.08	0.775
Male	556 (52.1%)	285 (52.6%)	271 (51.5%)		
Female	512 (47.9%)	257 (47.4%)	255 (48.5%)		
Boarding status (%)	100%	100%	100%	—	—
Height (cm)	169.2 ± 8.4	169.5 ± 8.1	168.9 ± 8.6	1.17	0.241
Weight (kg)	62.8 ± 10.5	62.2 ± 10.2	63.4 ± 10.8	1.87	0.048*
BMI (kg/m^2^)	21.9 ± 3.2	21.7 ± 3.1	22.1 ± 3.3	2.04	0.042*

Data are presented as mean ± SD or frequency (%). *p < 0.05. *t*-values and *p-*values were computed using independent samples *t*-tests (equal variances assumed). Although weight and BMI showed small but statistically significant differences between groups, these baseline differences were considered when interpreting subsequent association and mediation analyses.

The participant recruitment and exclusion process is summarized in Supplementary Table S1.

The study protocol was approved by the Medical Ethics Committee of Shenzhen Children’s Hospital. Written informed consent was obtained from all participants and their legal guardians before data collection. For the accelerometry validation sub-sample (*n* = 108), participants were purposively selected from students who exhibited a sedentary phenotype, while maintaining comparable representation across uniform groups and gender where possible. This sampling strategy was used to examine the correspondence between self-reported NEAT-related behaviors and accelerometry-derived activity metrics in a low-activity subgroup. All data collection was conducted during a standard mid-semester week to avoid the influence of extreme weather or examination-related stress on habitual physical activity.

### 2.3 Uniform classification and exposure

Participants were categorized based on the mandatory uniform policy of their respective schools.

*Sport uniform group*: schools requiring loose-fitting, elastic synthetic or cotton-blend tracksuits, characterized by high fabric extensibility and ergonomic designs that do not hinder large-scale limb movements.

*Formal uniform group*: schools requiring traditional “blazer-and-slacks/skirts” attire. These uniforms utilize non-elastic woven fabrics and tailored cuts, featuring stiff collars, waistbands, and restrictive skirts for females.

All participants were required to wear the full uniform for a minimum of 8.5 h per day (covering all academic sessions and self-study periods) during the five-day boarding week.

### 2.4 NEAT assessment: the multi-method approach

To approximate NEAT-related low-intensity behaviors of daily spontaneous movements, a dual-measurement strategy was implemented.

#### 2.4.1 Self-reported questionnaire

All 1,068 participants completed the Adolescent NEAT Questionnaire (ANQ), an adapted self-reported questionnaire developed to assess NEAT-related behaviors based on Levine’s NEAT framework (4), with item wording and response formatting informed by prior questionnaire-based physical activity assessment in Chinese children (25, 26). The questionnaire covered low-intensity non-exercise behaviors, including transit speed between classes, standing frequency, fidgeting habits, and informal movement during breaks. The item domains, response format, and scoring direction are provided in Supplementary Table S2. Additionally, the questionnaire included items assessing the frequency and duration of structured exercise to estimate baseline MVPA, which served as a covariate in subsequent analyses.

#### 2.4.2 Accelerometry-based calibration (10% sub-sample)

To compare self-reported ANQ scores with accelerometry-derived metrics, a sub-sample of 108 students (54 per uniform group, proportioned by gender and baseline BMI) wore tri-axial accelerometers for seven consecutive days. Data were sampled at 30 Hz (27). A specialized algorithm utilized institutional timetables to automatically filter out MVPA occurring during scheduled Physical Education (PE) classes and extracurricular sports (28). Only data between 1.0 and 3.0 Metabolic Equivalents (METs) (29) during non-exercise hours were utilized to calculate “Non-exercise Steps” and “Daily Sedentary Time.” In this sub-sample, the self-reported NEAT score showed a statistically significant difference between groups (*p* = 0.035), with moderate-to-strong correlations to objective measures (*r* = 0.72 for steps and *r* = −0.68 for sedentary time, both *p* < 0.01).

For this validation sub-study, a purposive sampling strategy was used to identify students with a sedentary phenotype based on 3-month historical WeChat Step data, defined as being consistently in the bottom quartile of daily steps and having no recorded extracurricular sports participation. This design was intended to reduce variation from voluntary sports participation and to focus on low-intensity non-exercise activity. Therefore, the accelerometry results should be interpreted as preliminary validation evidence within this sedentary sub-sample rather than as full validation of the ANQ in the entire cohort.

### 2.5 Anthropometric measurements

Height (cm) and weight (kg) were measured by trained health professionals using calibrated digital medical scales (Seca 877, Hamburg, Germany) (30). Participants were measured in light indoor clothing without shoes. BMI was calculated as weight (kg)/height (m)^2^. To ensure accuracy, measurements were taken twice and averaged, with a third measurement required if the difference exceeded 0.5 kg or 0.5 cm.

### 2.6 Data processing and quality control

Accelerometer data were considered valid if the device was worn for at least 10 h per day for a minimum of four weekdays and one weekend day. Automated data cleaning protocols were applied to detect “non-wear time” (defined as ≥60 min of consecutive zero counts) (31). For the questionnaire data, internal consistency was verified using Cronbach’s alpha (*α* = 0.84), and Mahalanobis distance was used to identify and exclude multivariate outliers.

### 2.7 Statistical analysis

Data were analyzed using SPSS version 26.0 (IBM Corp, Armonk, NY) and the PROCESS macro (v4.0) developed by Hayes and Rockwood (32). Prior to the main analysis, the normality of distribution was assessed using the Kolmogorov–Smirnov test, and all continuous variables, including NEAT scores and BMI, met the requirements for parametric testing. To examine preliminary convergent evidence for the self-reported NEAT-related questionnaire, Pearson correlation coefficients (r) were calculated between questionnaire scores and accelerometry-derived metrics within the sedentary validation sub-sample.

To examine the primary association between uniform type and BMI, unadjusted independent sample t-tests were conducted for the total sample and subsequently stratified by gender (as presented in Tables 1, 2). The magnitude of differences was quantified using Cohen’s d as an effect size (ES) metric, categorized as negligible (<0.2), small (0.2–0.5), medium (0.5–0.8), and large (>0.8). An interaction analysis (Uniform Type × Gender) was performed using two-way ANOVA to determine whether the association between uniform type and BMI was gender-dependent.

**Table 2:** Gender-specific association between uniform type and BMI.

Gender	Uniform type	n	BMI (mean ± SD)	Mean diff.	*p*-value	Cohen’s d (ES)
Male (boys)	Sport	285	22.5 ± 3.0	0.1	0.699	0.03 (Negligible)
	Formal	271	22.6 ± 3.1			
Female (girls)	Sport	257	20.8 ± 2.8	0.7	0.009	0.24 (Small)
	Formal	255	21.5 ± 3.2			

Mean differences were tested using independent samples *t*-tests. *p < 0.05, and **p < 0.01. Cohen’s d effect sizes: negligible (<0.2), small (0.2–0.5). A significant gender × uniform interaction was observed (*p* = 0.032, two-way ANOVA).

The mediation analysis (Model 4) and the moderated mediation analysis (Model 7) were conducted using the PROCESS macro to examine the statistical association pattern linking uniform type, NEAT-related behaviors, and BMI. In these models, Uniform Type (0 = Sport, 1 = Formal) was designated as the independent variable (X), NEAT Score as the mediator (M), and BMI as the dependent variable (Y). To reduce potential confounding of uniform type, the following covariates were controlled: Age, Height, and self-reported MVPA. Gender was included as a covariate in the total sample models but excluded from the gender-stratified analyses.

The mediation effect was tested using the non-parametric percentile bootstrap method with 5,000 resamples (33). A mediation effect was considered statistically significant if the 95% bias-corrected confidence interval (95% CI) did not include zero. Additionally, the Sobel test was employed to confirm the significance of the indirect path, and the Proportion Mediated (P_M) was calculated to quantify the explanatory power of NEAT. Significance for all tests was set at a two-tailed *α* = 0.05.

Given the cross-sectional design, the mediation and moderated mediation analyses were interpreted as statistical association patterns rather than evidence of causal pathways, as temporal ordering cannot be established in cross-sectional mediation models (34).

## 3 Results

**Figure 2:**
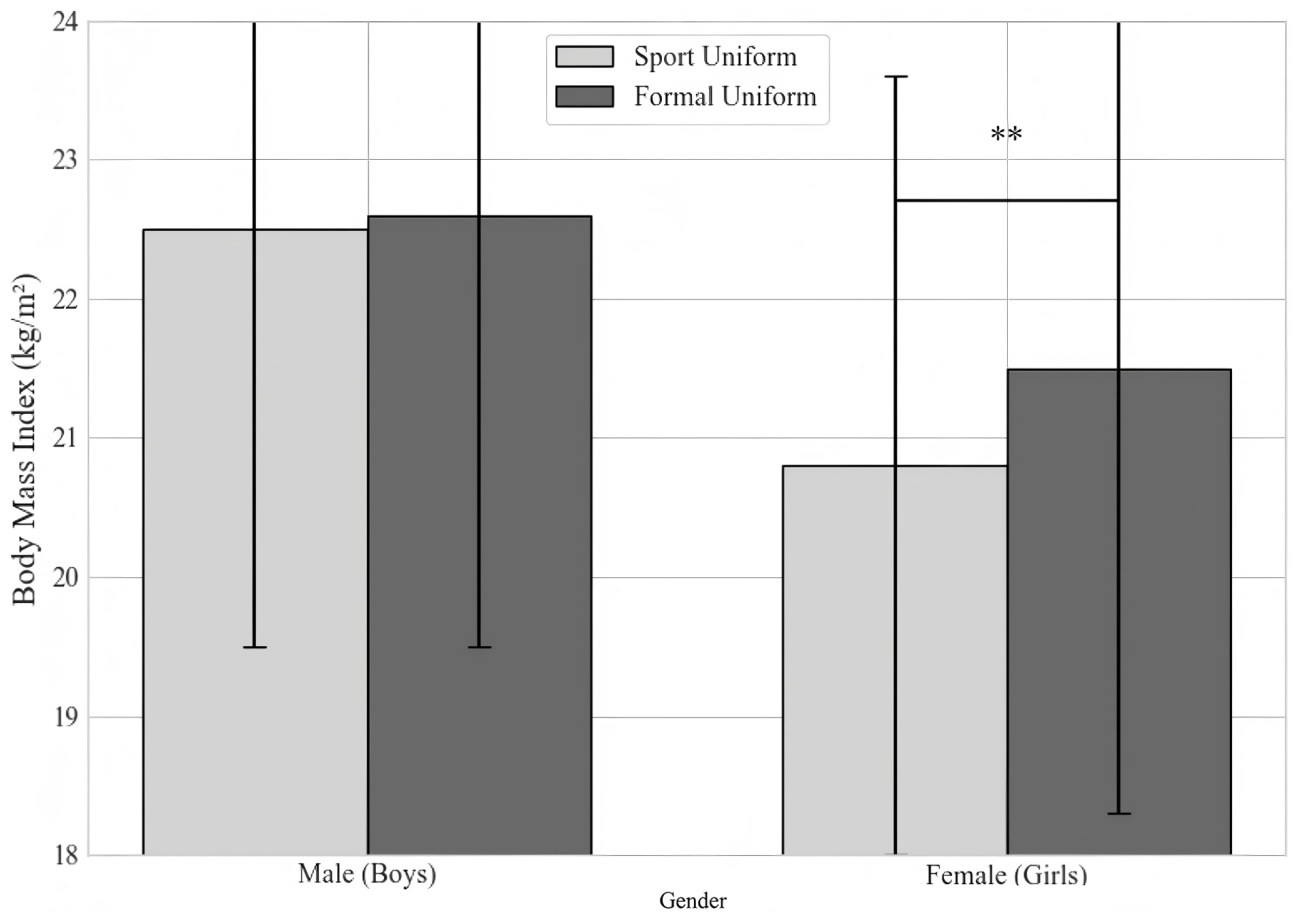
Gender-specific association between uniform type and BMI. The symbol ** in figure indicates *p* < 0.01.

In the accelerometer sub-sample (*n* = 108), self-reported NEAT scores were significantly lower in the formal uniform group than in the sport uniform group [58.2 ± 11.8 vs. 62.4 ± 12.5; mean difference = 4.2, 95% CI (1.1, 7.3), *p* = 0.035]. Objective accelerometry showed a similar pattern: formal uniform students accumulated 670 fewer non-exercise steps per day (5,580 ± 920 vs. 6,250 ± 980; *p* < 0.001) and spent 35 more minutes per day sedentary (595 ± 45 vs. 560 ± 40 min/day; *p* < 0.001). Within the sedentary validation sub-sample, self-reported NEAT-related scores showed preliminary convergent evidence with accelerometry-derived measures (r = 0.72 for non-exercise steps and r = −0.68 for sedentary time, both *p* < 0.01; Table 3). These findings support the use of the ANQ as an approximate indicator of NEAT-related behaviors in the present study, although they should not be interpreted as full psychometric validation in the entire sample. A significant gender × uniform type interaction was observed in a two-way ANOVA (*F* = 4.62, *p* = 0.032), indicating that the association between uniform type and BMI differed by gender. Stratified analyses showed no significant difference in BMI between uniform groups among boys (22.6 ± 3.1 vs. 22.5 ± 3.0 kg/m^2^; mean difference = 0.1, *p* = 0.699, Cohen’s d = 0.03). In contrast, girls wearing formal uniforms had significantly higher BMI than those wearing sport uniforms (21.5 ± 3.2 vs. 20.8 ± 2.8 kg/m^2^; mean difference = 0.7, *p* = 0.009, Cohen’s d = 0.24; see Table 2 for additional analysis). The gender-specific disparity in BMI and the subsequent non-significant findings among boys compared to the significant elevation among girls are visually summarized in Figure 2. Gender-specific association between uniform type and BMI.

**Table 3:** Preliminary comparison of self-reported NEAT and accelerometry in the sedentary sub-sample.

Outcomes	Sport uniform (*n* = 54)	Formal uniform (*n* = 54)	Mean diff. (95% CI)	*p*-value	Correlation w/survey (r)
NEAT score (survey)	62.4 ± 12.5	58.2 ± 11.8	4.2 (1.1, 7.3)	0.035*	—
Non-exercise steps	6,250 ± 980	5,580 ± 920	670 (310, 1,030)	<0.001***	0.72
Sedentary time (min/d)	560 ± 40	595 ± 45	−35 (−55, −15)	<0.001***	−0.68

Accelerometer data were collected over 7 consecutive days; non-exercise steps exclude organized sports sessions. *p < 0.05, **p < 0.01, ***p < 0.001.

Correlations (r) indicate preliminary convergent evidence between self-reported NEAT-related scores and accelerometry-derived metrics within the sedentary validation sub-sample. These results should not be interpreted as full psychometric validation of the ANQ in the entire cohort.

**Figure 3:**
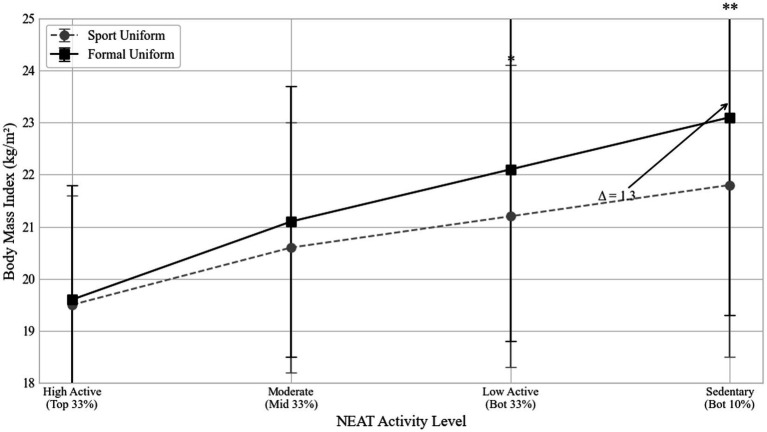
Exploratory subgroup pattern by NEAT-related activity level. *p < 0.05, and **p < 0.01.

The exploratory subgroup pattern across NEAT-related activity levels is illustrated in Figure 3. This analysis was used to examine whether the uniform-BMI association differed by baseline activity level.

The association between formal uniforms and BMI appeared larger among girls with lower spontaneous activity levels. No significant difference was observed in the high-active tertile (top 33% NEAT: mean difference = +0.1 kg/m^2^, *p* = 0.835). The observed BMI difference increased in the moderate-active tertile (+0.5 kg/m^2^, *p* = 0.065) and became statistically significant in the low-active tertile (bottom 33% NEAT: +0.9 kg/m^2^, *p* = 0.015). The largest observed difference was found in the most sedentary decile (bottom 10% NEAT: +1.3 kg/m^2^, *p* = 0.004; see Table 4 for stratified results). Mediation analysis using PROCESS Model 4 with 5,000 bootstrap resamples indicated that NEAT-related behaviors were statistically consistent with mediation in the association between uniform type and BMI in the female subsample (*n* = 512; see Table 5, Panel A for mediation analysis). Uniform type was associated with lower NEAT-related scores (a = −4.52, *p* < 0.001), and lower NEAT-related scores were associated with higher BMI (b = −0.03, *p* < 0.001). The total effect of uniform type on BMI was significant (c = 0.20, *p* = 0.013). After including NEAT, the direct effect became non-significant [c′ = 0.08, *p* = 0.254; 95% CI (−0.06, 0.22)], while the indirect effect was significant [ab = 0.12, 95% bootstrap CI (0.05, 0.21)], accounting for 60.3% of the total effect.

**Table 4:** Exploratory subgroup analysis by NEAT-related activity level among female students.

Activity level (NEAT quantiles)	Sport group BMI (n)	Formal group BMI (n)	Difference (*Δ*)	*t*-value	*p*-value
High active (top 33%)	19.8 ± 2.2	19.9 ± 2.3	0.1	0.21	0.835
Moderate active (mid 33%)	21.1 ± 2.5	21.6 ± 2.7	0.5	1.85	0.065
Low active (bottom 33%)	21.9 ± 3.1	22.8 ± 3.4	0.9	2.45	0.015*
Sedentary (bottom 10%)	22.4 ± 3.5	23.7 ± 4.0	1.3	2.98	0.004**

Participants were stratified into tertiles (top, middle, bottom 33%) and the bottom decile (10%) based on NEAT scores in the female subsample. *p < 0.05, and **p < 0.01. Differences were tested using independent samples *t*-tests within each stratum. The bottom-decile analysis was exploratory and should be interpreted as a subgroup pattern rather than a confirmatory causal effect.

**Table 5:** Mediation and moderated mediation analysis.

Panel A: mediation analysis (female subsample, *n* = 512)						
Path/effect	Coeff. (β)	SE	*t*-value	*p*-value	95% CI (Bootstrap)	Sobel Z
Total effect (c)	0.204	0.082	2.49	0.013*	[0.042, 0.366]	—
Direct effect (c’)	0.081	0.071	1.14	0.254	[−0.058, 0.220]	—
Indirect effect (ab)	0.123	0.038	—	—	[0.054, 0.208]	3.18
Proportion mediated (P_M)	60.30%	—	—	—	—	—

Panel B: test of moderated mediation (gender as moderator)				
Conditional indirect effects	Effect	Boot SE	95% CI	Conclusion
Male (boys)	0.012	0.015	[−0.018, 0.045]	Not Significant
Female (girls)	0.123	0.038	[0.054, 0.208]	Significant

Index of moderated mediation	Diff	Boot SE	95% CI	Inference
Gender × Uniform → NEAT	0.111	0.042	[0.035, 0.198]	Statistically supported

Panel A, Mediation analysis (PROCESS Model 4, females only, *n* = 512) with 5,000 bootstrap resamples. Panel B, Moderated mediation (PROCESS Model 7, gender as moderator of the a-path). ***p < 0.001. Significant moderated mediation index indicates gender-dependent indirect effect.

Because the study was cross-sectional, these estimates should be interpreted as statistical mediation patterns rather than causal mediation effects.

Moderated mediation analysis using PROCESS Model 7 indicated that gender moderated the indirect effect of uniform type on BMI via NEAT (see Table 5, Panel B for moderated mediation results). The index of moderated mediation was statistically significant [Index = 0.111, 95% bootstrap CI (0.035, 0.198)]. The conditional indirect effect was significant among girls [0.123, 95% bootstrap CI (0.054, 0.208)] but non-significant among boys [0.012, 95% bootstrap CI (−0.018, 0.045)].

In summary, formal school uniforms were associated with lower NEAT-related scores and higher BMI among adolescent girls in this boarding school setting, particularly among those with low spontaneous activity levels. This association was consistent with a statistical mediation pattern through NEAT-related behaviors and was not observed among boys, suggesting a gender-specific association between formal uniform use and BMI in this boarding school sample.

## 4 Discussion

The present study provides novel empirical evidence linking school uniform policies to adolescent body composition within a strictly controlled 100% boarding environment. Our findings suggest that formal uniforms were associated with higher BMI, particularly among female students, and that this association was statistically consistent with mediation through NEAT-related behaviors. By focusing on a 100% boarding school setting, this study reduced variability related to diet, commuting, and daily schedules, while acknowledging that residual confounding could not be eliminated. A pivotal finding of this study is the significant Gender × Uniform interaction, which aligns with the “Environmental Press” hypothesis. Females in formal attire exhibited a higher BMI. This asymmetry suggests that traditional female uniforms may represent both mechanical and psychosocial constraints: mechanical restriction and psychological inhibition (10, 16). From a mechanical point of view, the range of motion required for spontaneous movements, such as taking longer steps, dynamic sitting, or stretching, can be limited by the structural rigidity and lack of elasticity inherent in formally woven fabrics (11). Furthermore, biomechanical analyses indicate that skirt-based ensembles significantly reduce step length and hip flexion angles compared to trousers during habitual walking, thereby subtly increasing the metabolic cost of movement initiation (35). The findings provided partial support for H1, as the uniform-BMI association was observed among female students but not among male students; supported H2 as a statistical mediation pattern; and offered exploratory support for H3 through the subgroup pattern observed among students in the lowest NEAT-related activity decile.

From a psychosocial perspective, clothing-related modesty norms and self-surveillance may partly explain why the observed association was more apparent among female students. However, psychological constructs such as postural anxiety, self-objectification, and perceived clothing discomfort were not directly measured in this study. Therefore, these explanations should be interpreted as theoretical interpretations rather than empirically tested mediators (18, 36). These theoretical perspectives suggest that clothing-related self-surveillance may be associated with reduced freedom of movement (37, 38). In contrast, the “sport uniform” (tracksuits), which is culturally gender-neutral and functionally elastic, may reduce perceived barriers to spontaneous movement.

These interpretations should not be generalized to all female adolescents, as clothing norms, uniform design, and school activity culture may vary across settings.

The mediation analysis suggested that the observed association between uniform type and BMI was statistically consistent with mediation through NEAT-related behaviors. The objective accelerometry data revealed a daily deficit of approximately 670 steps and an increase of 35 min in sedentary time among the formal uniform group. While a daily deficit of ~670 steps may appear trivial in the short term, the “cumulative energy imbalance” theory suggests otherwise (22). Pediatric energy-gap analyses suggest that a persistent “energy gap” of as little as 15–50 kcal/day can lead to substantial weight gain over extended periods (39). Over the course of a 3-year boarding school tenure, this minor but persistent lower activity-related energy expenditure may contribute to a positive energy balance and partly account for the observed BMI differences, a phenomenon consistent with the “creeping obesity” observed in restrictive environments (40). This finding suggests that NEAT-related behaviors may complement MVPA-focused perspectives when examining daily activity patterns in school settings (4, 6).

The subgroup analysis suggested a possible vulnerability pattern among students with lower NEAT-related activity levels. In this subgroup, the uniform-BMI difference appeared larger, suggesting that students with fewer spontaneous activity opportunities may be more sensitive to environmental constraints. Because this analysis was exploratory, the finding should be interpreted cautiously and confirmed in longitudinal studies (41–43).

Several limitations should be acknowledged. First, the cross-sectional design precludes causal inference and cannot establish the temporal ordering among uniform type, NEAT-related behaviors, and BMI. Reverse causality also cannot be excluded, as higher BMI may be associated with lower spontaneous activity. Second, although the ANQ showed acceptable internal consistency in this sample, test–retest reliability and factor structure were not examined, and its comparison with accelerometry was limited to the sedentary validation sub-sample. Because the accelerometry sub-sample was purposively selected from students with a sedentary phenotype, the observed correspondence between uniform type and activity metrics may be stronger than would be expected in a more representative full-sample validation. Third, psychological constructs such as postural anxiety, self-objectification, and perceived clothing discomfort were not directly measured; therefore, the proposed psychosocial pathway should be regarded as a theoretical interpretation rather than an empirically tested mechanism. Fourth, although the 100% boarding school context helped reduce variation in diet, commuting, and daily schedules, potential confounders such as detailed dietary intake, socioeconomic status, sleep patterns, pubertal stage, and school-specific recreational policies were not fully assessed. Finally, the findings were based on boarding schools and may not be generalizable to day schools or other educational contexts.

Nevertheless, the observed uniform-NEAT association should be interpreted as indirect behavioral evidence rather than as direct proof that formal uniforms reduce NEAT.

## 5 Conclusion

This study suggests that formal school uniforms, particularly those involving skirts and non-elastic fabrics, were associated with higher BMI among female adolescents in this boarding school sample. This association was statistically consistent with mediation through lower NEAT-related behaviors. Exploratory subgroup analyses further suggested that the uniform-BMI association was more apparent among students with lower NEAT-related activity levels. These findings suggest that school uniform design may be a relevant environmental factor for further investigation in adolescent health research. From a practical perspective, movement-friendly uniform designs may deserve consideration in residential school settings, although longitudinal or intervention-based studies are needed before making causal or policy conclusions. Future studies should examine whether changes in school uniform design can meaningfully influence NEAT-related behaviors and BMI trajectories over time.

## Data Availability

The raw data supporting the conclusions of this article will be made available by the authors, without undue reservation.
