# Supplementary material for: Formal uniforms as an environmental constraint on NEAT and adolescent obesity: a cross-sectional study in boarding schools
Source: Front Public Health. 2026 Jun 12;14:1817702. doi: 10.3389/fpubh.2026.1817702 (PMC13303609; doi:10.3389/fpubh.2026.1817702)
Supplement: Supplementary file 1 [file Table_1.docx]

**Supplementary Table S1.** Participant Recruitment and Exclusion Process

| **Recruitment stage** | **n** |
| --- | --- |
| Initially recruited students | 1,200 |
| Excluded due to chronic metabolic disorders, movement-limiting disabilities, or incomplete questionnaires | 132 |
| Final analytic sample | 1,068 |
| Sport uniform group | 542 |
| Formal uniform group | 526 |
| Accelerometry validation sub-sample | 108 |
