# Supplementary material for: Formal uniforms as an environmental constraint on NEAT and adolescent obesity: a cross-sectional study in boarding schools
Source: Front Public Health. 2026 Jun 12;14:1817702. doi: 10.3389/fpubh.2026.1817702 (PMC13303609; doi:10.3389/fpubh.2026.1817702)
Supplement: Supplementary file 2 [file Table_2.docx]

**Supplementary Table S2.** Item Domains, Response Format, and Scoring Guide for the Adolescent NEAT Questionnaire (ANQ)

| **Item No.** | **Domain** | **Operational focus** | **Item stem** | **Response options** | **Scoring** |
| --- | --- | --- | --- | --- | --- |
| ANQ-1 | Between-class movement | Walking pace during school transitions | During classroom or building transitions, I walked at a pace faster than slow strolling. | 0 = never; 1 = 1–2 times/week; 2 = 3–4 times/week; 3 = once/day; 4 = several times/day | Higher = more NEAT-related movement |
| ANQ-2 | Between-class movement | Voluntary walking during breaks | During short breaks, I walked around rather than remaining seated. | 0–4 frequency scale | Higher = more NEAT-related movement |
| ANQ-3 | Between-class movement | Additional informal walking | I took additional short walks on campus outside required classroom transitions. | 0–4 frequency scale | Higher = more NEAT-related movement |
| ANQ-4 | Stair use | Stair preference during routine movement | When stairs were available, I used stairs during routine school movement. | 0–4 frequency scale | Higher = more NEAT-related movement |
| ANQ-5 | Standing behavior | Standing during non-class periods | During breaks or waiting periods, I stood rather than sat. | 0–4 frequency scale | Higher = more NEAT-related movement |
| ANQ-6 | Standing behavior | Accumulated standing episodes | I had repeated short standing episodes outside class time. | 0–4 frequency scale | Higher = more NEAT-related movement |
| ANQ-7 | Postural transitions | Changing body position | I changed posture frequently, such as standing up, sitting down, leaning, or shifting position. | 0–4 frequency scale | Higher = more NEAT-related movement |
| ANQ-8 | Postural transitions | Dynamic sitting | While seated, I adjusted my posture or shifted body position. | 0–4 frequency scale | Higher = more NEAT-related movement |
| ANQ-9 | Fidgeting behavior | Awareness of small spontaneous movements | I noticed myself making small movements, such as tapping my feet, moving my legs, or changing hand position. | 0–4 frequency scale | Higher = more NEAT-related movement |
| ANQ-10 | Fidgeting behavior | Minor movement during study periods | During self-study or seated learning periods, I noticed small body movements rather than remaining completely still. | 0–4 frequency scale | Higher = more NEAT-related movement |
| ANQ-11 | Informal light activity | Light activity during breaks | During breaks, I engaged in light informal activities such as walking with classmates or casual movement-based play. | 0–4 frequency scale | Higher = more NEAT-related movement |
| ANQ-12 | Informal light activity | Movement outside structured exercise | Outside PE classes or organized sports, I chose light physical movement when possible. | 0–4 frequency scale | Higher = more NEAT-related movement |
| ANQ-13 | Sedentary tendency | Prolonged uninterrupted sitting | During non-class time, I remained seated for long periods without standing or walking. | 0–4 frequency scale | Reverse scored |
| ANQ-14 | Sedentary tendency | Avoidance of unnecessary movement | I avoided unnecessary walking or standing during the school day. | 0–4 frequency scale | Reverse scored |
| ANQ-15 | Sedentary tendency | Preference for stillness | I preferred to remain physically still whenever school rules or schedules allowed. | 0–4 frequency scale | Reverse scored |

**Note:** Items were rated for the past 7 school days on a 0–4 frequency scale, excluding PE classes, organized sports, and off-campus weekend activities. ANQ-13–15 were reverse scored. Total scores ranged from 0 to 60 and were standardized to a 0–100 scale, with higher scores indicating more frequent NEAT-related behaviors. Cronbach’s α was 0.84. The ANQ was used as an approximate behavioral index rather than a direct measure of caloric expenditure or psychological constructs; accelerometry comparison was limited to the sedentary validation sub-sample.
